# Rooming-in organization to prevent neonatal mortality and morbidity in late preterm infants

**DOI:** 10.1186/1824-7288-40-S2-A4

**Published:** 2014-10-09

**Authors:** Mariano Manzionna, Antonio Di Mauro

**Affiliations:** 1 Pediatric Unit, Maternal and Child Health Department, S. Giacomo Hospital, ASL BA, Monopoli (Bari), Italy; 2 Neonatology and Neonatal Intensive Care Unit, Department of Biomedical Science and Human Oncology, University of Bari “Aldo Moro”, Bari, Italy

## 

Despite most infants born at 34+0 through 36+7 weeks’ gestation are thought to be at low risk during the birth hospitalization and have a neonatal course with no significant complications, they are physiologically and metabolically immature with an higher rates of morbidity and mortality than term infants [1].

Most common medical condition associated with late-preterm births are respiratory distress, apnea, temperature instability, hypoglycemia, hypocalcemia, jaundice, poor feeding, sepsis and finally an higher rates of the hospital readmissions during the neonatal period. These morbidities result in workup for sepsis evaluations, antibiotic therapy, intravenous fluid administration, ventilatory support and increased length of stay with higher hospital costs [2].

Rooming-in organization of late preterms births aims to assess and identify risk factors, prevent and manage potential medical complications during hospitalization. Interventions and practices reccomended are illustred in table 1.

**Table 1:** Assessment and care of the late preterm infant [3].

Assess gestational age of neonate Assess and monitor respiratory status Appropriate respiratory interventions Assess for risk factors and symptoms of heat loss and/or cold stress Interventions to maintain a neutral thermal environment Interventions and assessment of hypoglycemia including transfer to higher acuity unit or facility if indicated Assess for maternal and neonatal risk factors for sepsis Antibiotic therapy and diagnostic evaluation if sepsis is suspected Assess for presence of jaundice and hyperbilirubinemia Phototherapy as indicated Parent education regarding signs and symptoms of jaundice and hyperbilirubinemia Breastfeeding, and support for breastfeeding mothers including observation, education and validation Discharge planning including parent education, counseling, and validation of knowledge about recognizing and acting on risk factors

Evidence of physiologic maturity, feeding competency, thermoregulation and absence of medical of medical illness are minimum discharge criteria for late-preterm newborns. Furthermore it’s of great importance to assess educational programs with special instruction and guidance to parents, engaging families in providing appropriate home care after hospital discharge. A long term follow-up arrangements is also recommended to assess and plan early interventions in case of neurodevelopment delay [4].

We conclude that, based on the significant morbidity and mortality of late preterm births, the health care focus on prematurity should be expanded to include the late preterm period.
